# Genomic Prediction and Selection for Fruit Traits in Winter Squash

**DOI:** 10.1534/g3.120.401215

**Published:** 2020-08-19

**Authors:** Christopher O. Hernandez, Lindsay E. Wyatt, Michael R. Mazourek

**Affiliations:** Plant Breeding and Genetics Section, School of Integrative Plant Science, Cornell University, Ithaca, NY

**Keywords:** Genomic Prediction, Genomic selection, Genetic gain, Index selection, Horticultural crops, Fruit quality, GBLUP, Cucurbits, Squash, GenPred, Shared data resources

## Abstract

Improving fruit quality is an important but challenging breeding goal in winter squash. Squash breeding in general is resource-intensive, especially in terms of space, and the biology of squash makes it difficult to practice selection on both parents. These restrictions translate to smaller breeding populations and limited use of greenhouse generations, which in turn, limit genetic gain per breeding cycle and increases cycle length. Genomic selection is a promising technology for improving breeding efficiency; yet, few studies have explored its use in horticultural crops. We present results demonstrating the predictive ability of whole-genome models for fruit quality traits. Predictive abilities for quality traits were low to moderate, but sufficient for implementation. To test the use of genomic selection for improving fruit quality, we conducted three rounds of genomic recurrent selection in a butternut squash (*Cucurbita moschata*) population. Selections were based on a fruit quality index derived from a multi-trait genomic selection model. Remnant seed from selected populations was used to assess realized gain from selection. Analysis revealed significant improvement in fruit quality index value and changes in correlated traits. This study is one of the first empirical studies to evaluate gain from a multi-trait genomic selection model in a resource-limited horticultural crop.

All squash belong to the *Cucurbitaceae* family, which contains a number of small-genome, vining crops primarily grown for their fruit and seed (Ferriol and Picó 2008; Sun *et al.* 2017). Five squash species are recognized as domesticated: *Cucurbita moschata*, *Curcurbita pepo*, *Cucurbita maxima*, *Cucurbita argyrosperma*, and *Cucurbita ficifolia* (Ferriol and Picó 2008). Squash can be classified as either winter or summer squash depending on the stage of maturity at which they are consumed; summer squash are consumed immature, whereas winter squash are consumed at physiological maturity or later (Loy 2004). Improving fruit quality is a major breeding goal in winter squash (Paris 1994; Loy 2012). Butternut squash is a popular winter squash market class from the species *C. moschata*, known for its high fruit quality (Loy 2012). The *C. moschata* genome has been published and quantitative trait loci (QTL) have been identified for a number of important fruit quality traits including carotenoid and free sugar content (Montero-Pau *et al.* 2017; Zhong *et al.* 2017). QTL mapping has led to a better understanding of fruit quality genetic variation in squash; however, using only markers associated with mapped QTL does not provide a practical means of improving quantitative traits in breeding programs (Bernardo and Yu 2007). Methods that can leverage all available marker data will likely be more effective at improving complex traits in squash.

According to the infintesimal model, quantitative traits are conditioned by many loci of small effect (Falconer and Mackay 1996). Small-effect QTL are difficult and expensive to map, are usually population-specific, and the effects of QTL that manage to pass significance thresholds are often overestimated (Beavis effect) (Bernardo 2016; Xu 2003). Advances in DNA sequencing technologies make high-density, affordable marker data a reality and have spurred innovation in marker-assisted breeding (Buckler *et al.* 2016). Genomic selection (GS) has emerged as a promising method for leveraging these data for the improvement of quantitative traits. In GS, whole-genome markers are used to capture the effects of all QTL (Meuwissen *et al.* 2001). Marker effects are estimated using statistical modeling in a training population, *i.e.*, a population that is phenotyped, genotyped and closely related to breeding material. After model training, the breeding value (BV) of selection candidates can be estimated from marker data alone (Meuwissen *et al.* 2013). BVs estimated from genomic selection models are referred to as genomic estimated breeding values (GEBVs) to distinguish them from BVs estimated using other methods. Unlike earlier marker-based approaches, GS need not employ significance testing or trait mapping.

Genomic Best Linear Unbiased Prediction (GBLUP) is one of the most widely-used statistical modeling approaches for GS (Heslot *et al.* 2015). GBLUP utilizes a mixed linear model (MLM), commonly represented in matrix notation as y=Xβ+Zμ+e, to obtain GEBVs. In this framework, GEBVs are modeled as random effects (*μ*) that are multivariate normally distributed $\mu ∼N\left(0,K\sigma_{\mu }^{2}\right)$ (Endelman 2011). The matrix K is a marker-based relationship matrix, which provides an estimate of the relationship between all individuals included in the analysis and enables prediction through information sharing among relatives (Habier *et al.* 2007). There are many advantages to GBLUP including computational efficiency and its compatibility with existing MLM methodology and software (Wang *et al.* 2018; Mrode 2014). Notably, GBLUP models are easily extended from using information from single traits (univariate) to integrating multiple traits (multivariate) (Jia and Jannink 2012). This is appealing because improving multiple traits is usually necessary to meet breeding goals (Hallauer and Miranda 1981).

Multi-trait selection is complicated by the fact that traits are rarely independent and frequently differ in economic importance (Smith 1936). One of the most efficient methods for multi-trait selection is index selection (Hazel and Lush 1942). In index selection, an equation is used to combine information from multiple traits, yielding a single value for selection (Baker 1986). Many different indices have been suggested including indices based on economic value, genetic correlations, phenotypic correlations, and improving some traits while restricting movement in other traits (Baker 1986). The Smith-Hazel index draws on all major sources of information: economic, phenotypic, and genetic (Hazel 1943). Multi-trait GBLUP (MT-GBLUP) simplifies the use of comprehensive indices, such as the Smith-Hazel index, as the optimal weights for the GEBVs obtained from MT-GBLUP are breeding program-defined economic weights (Mrode 2014; Okeke *et al.* 2017; Jia and Jannink 2012).

Winter squash are amenable to many breeding methods, but most are difficult to implement due to space restrictions (Loy 2012). Individual squash plants can reach several meters in radius, making large populations infeasible. On top of this, fruit quality traits cannot be accurately phenotyped until long after flowering—limiting selection to the female parent in most scenarios (Loy 2004). Further, it can be difficult to both obtain accurate fruit quality estimates and perform controlled pollination, as fruit culling is practiced until a successful pollination is achieved. This reduces the number of fruit that can be used for fruit quality estimation and distorts yield data. These restrictions limit breeding population size, the use of greenhouse generations, the accuracy of selection, and, ultimately, genetic gain. GS could potentially disburden the improvement of fruit quality in squash by enabling selection prior to flowering, and thus, selection on both parents. With GS it would also be possible to screen hundreds of seedlings for fruit quality traits in a greenhouse, which is impossible with traditional methods. This would allow two cycles of selection per year: one in the field and one in the greenhouse. Simulation and empirical selection studies have demonstrated the superiority of GS over other methods for short-term gain in quantitative traits in cereal crop species (Bernardo and Yu 2007; Beyene *et al.* 2015; Massman *et al.* 2013; Combs and Bernardo 2013; Rutkoski *et al.* 2015). However, few studies have explored the use of GS for improving resource-limited horticultural crops, such as winter squash.

GS presents an attractive alternative for improving squash fruit quality. Few studies have explored the use of whole-genome prediction in horticultural systems (Wu *et al.* 2019; Würschum *et al.* 2013; Muranty *et al.* 2015; Iwata *et al.* 2013); even fewer have considered the impact of selecting on multiple traits simultaneously (Yamamoto *et al.* 2016; Covarrubias-Pazaran *et al.* 2018), and, to our knowledge, there have been no published GS studies involving multiple cycles of selection in horticultural crops. In this study we set out to evaluate whole genome models for an array of fruit traits, estimate important genetic and phenotypic parameters, and determine the impact of several rounds of GS on key fruit quality traits in a breeding population.

## Materials and Methods

### Genetic material

**Figure 1:**
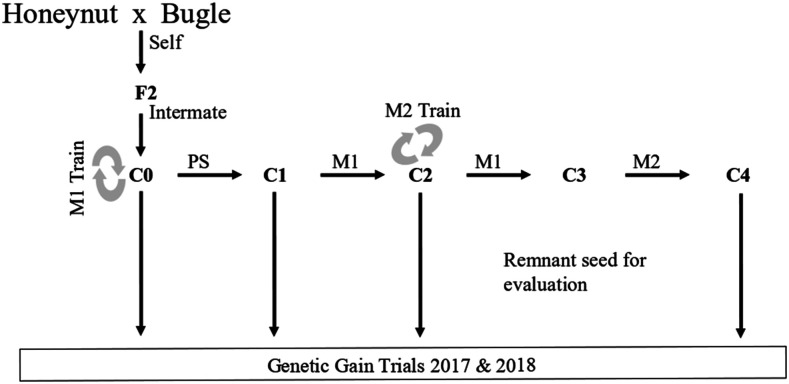
The base population (C0) was created from a randomly mated F2 population and was subjected to one cycle of phenotypic selection (PS) followed by three cycles of genomic selection. Two different genomic selection models, which are referred to as M1 and M2, were used for selection. Model training was accomplished during field generations (C0 and C2) when both phenotypic and genomic data were available and is designated with circular arrows in the schema. Remnant seed was used to evaluate gain from selection in genetic gain trials.

The base population was formed by crossing two Cornell pureline cultivars, Honeynut and Bugle (Hultengren *et al.* 2016). The resulting F1 was self pollinated to form the F2 generation. A population of around 60 F2 plants was grown in a greenhouse during the winter of 2014 and plants were randomly mated using at least three randomly selected male flowers to pollinate each fruit. Resulting seed was bulked, taking equal amounts from each half-sib family, and used to form the base population (see Figure 1). The base population will be referred to as the cycle 0 population (C0), and selection populations are numbered sequentially (C1, C2, C3 etc.). A genotyped and phenotyped population derived from the same cross as the base population, referred to as the test population (T1), was also used. The T1 population was originally developed for use in a separate breeding experiment that was not conducted, and data from this population were included in this study solely for cross validation and parameter estimation as described in subsequent sections.

### Genotypic data

Genomic DNA was extracted from young leaf tissue of selection candidates at first true leaf stage using the Qiagen DNeasy 96 Plant Kit (Qiagen, Valencia, CA, USA). Genotyping-by-sequencing (GBS) libraries were prepared with ApeKI, following a GBS protocol optimized for the squash genome (Holdsworth *et al.* 2016). The base population was genotyped in 2014 by preparing two 96-plex libraries and sequencing on two lanes of the Illumina HiSeq 2500 at the Weill Cornell Medical College Genomics Facility. In subsequent generations, 192-plex libraries were prepared by the University of Wisconsin Madison Biotechnology Center DNA Sequencing Facility and sequenced in one lane of the NextSeq 500 at the Cornell University Genomics Facility. Raw sequencing data were processed using the TASSEL-GBS pipeline (Glaubitz *et al.* 2014). SNP calling and filtering procedures varied depending on available resources and purpose, as described below.

SNP calling for selection was facilitated by a draft, pre-publication version of the *C. moschata* genome (Sun *et al.* 2017). A custom Python script was used to concatenate small scaffolds together to form a psuedomolecule for alignment. The first 19 largest scaffolds in the draft genome were kept as is and remaining scaffolds were concatenated with a padding of 80 “N”s to prevent read alignments to artificially adjacent segments. SNPs were then filtered using TASSEL (Bradbury *et al.* 2007) and VCFtools (Danecek *et al.* 2011) according to the following criteria: minor allele frequency (MAF) over 0.05, biallelic, and present in 80% or more of the selection candidates. Genotypes that were not supported by a read depth of at least two were set to missing. Individuals missing more than 40% of the markers were discarded. Marker data were transformed to a dosage matrix using either the ”–012” argument in VCFtools or using the atcg1234() function in the ”Sommer” R package (Covarrubias-Pazaran 2016). The ”rrBLUP” R package (Endelman 2011) function A.mat() was used with the ”EM” method for marker imputation and to convert the dosage matrix to an additive genetic relationship matrix.

SNP calling for genetic parameter estimation and cross-validation was achieved using all available data from the selection experiment, the latest version of the published *C. moschata* genome (Sun *et al.* 2017), and available sequencing information from the parents of the population. SNPs were filtered using similar criteria as those used for the selection experiment, except minimium read depth was set to seven, the depth at which TASSEL requires two reads to call a heterozygote, and sequencing information from the parents was incorporated. Only SNPs that were homozygous within parents but different between the parents, had 50% missing data or less, maximum heterozygousity less then 90%, and MAF ≥0.05 were retained. SNP sets were merged on common markers for each cross validation scenario/parameter estimation and LinkImpute (Money *et al.* 2015), as implemented by the TASSEL ”LDKNNiImputationHetV2Plugin” plugin, was used to impute missing data after removing indels. After imputation, data were re-filtered for less than 20% missing data, ≥0.05 MAF, and individuals missing more than 40% of data were removed. Any missing data left after this process were mean imputed. The relationship matrix was obtained using the same method used for selection.

### Phenotypic data

Plants were hand-harvested by placing all fruit from a plant into a labeled mesh bag. Fruit were then cured under standard conditions. Barcodes were used to keep track of single-fruit data. Each fruit was weighed and then sliced lengthwise to facilitate measurement of length, width, and flesh color. Length (Len) and width (Wd) were measured using a barcoded ruler (Mazourek 2017). Color of fruit flesh was determined using a Konica Minolta CR-400 Chromo Meter colorimeter with standard illuminant set to D65 and a 2${}^{\circ }$standard observer. The instrument was calibrated at the start of each day using a white point calibration tile, and raw XYZ colorspace values resulting from the average of three measurements were converted to CIE L* a* b* color space values. Slices of tissue were sampled from the neck portion of the fruit for percent dry matter (%DM) determination and Brix (${}^{\circ }$Bx) measurement. For %DM, approximately fifteen to thirty grams of tissue was weighed and then re-weighed after drying for about 24 hr in an Excalibur 9-tray food dehydrator set to 155${}^{\circ }$F. Another slice was frozen in a plastic bag at -20 ${}^{\circ }$C overnight, thawed the next day, and then squeeze-juiced for ${}^{\circ }$Bx determination using an ATAGO PAL-1 pocket refractometer. Plant yield was measured in three different ways: number of fruit per plant (FrtCt), total weight of all fruit per plant (TotalWt), and total weight adjusted for average percent dry matter (TotalDM).

### Breeding scheme and evaluation

Four sequential rounds of selection were practiced from C0: one round of phenotypic selection, followed by three rounds of GS using an index derived from an MT-GBLUP model. The various field sites are described in Table 1. Population size was around 200 plants in each generation. The breeding scheme is shown in Figure 1, and each round of selection is described below. GS models were trained at two different points. C0 was used to train the initial model for selection (M1), and data from C2 was used to train a new model (M2) for the last round of selection.

**Table 1:** A description of field sites

Site Code	Site Name	Location	Year	Management
Field-EI1	East Ithaca	42.4413493,-76.4733234	2014, 2017	Transitional Organic
Field-EI2	East Ithaca	42.440784,-76.4713935	2015, 2016	Transitional Organic
Field-Fr1	Homer C. Thompson Vegetable Research Farm	42.521724,-76.3347793	2017	Conventional
Field-Fr2	Freeville Organic Research Farm	42.523819,-76.327854	2017	Certified Organic
Field-Fr3	Homer C. Thompson Vegetable Research Farm	42.517705,-76.3346863	2018	Conventional

***Selection from Cycle 0*** Selections from Cycle 0 were based on single-plant phenotypes using criteria commonly employed in our squash breeding field program. Briefly, twenty plants, corresponding to 10% of the population, were picked such that they were in the top 20% for ${}^{\circ }$Bx, % DM, and $a^{\text{*}}$ color value but not in the bottom 50% for yield.

***Selection in Cycle 1*** Equal amounts of seed from plants phenotypically selected from C0 were planted in 72-cell trays in the greenhouse and genotyped with GBS at the first true leaf stage,these plants constituted the C1 population. The top 10% of the C1 population was then selected based on an index value calculated from the MT-GBLUP model. Selected seedlings were transplanted to larger pots and were randomly mated to produce the C2 seed. To achieve random mating in the greenhouse, all male flowers were picked on the mornings that female flowers were open. Each female flower was then pollinated by 4-5 random male flowers by dabbing pollen from males on different sectors of the stigma.

***Selection in Cycles 2 & 3*** As in selection in C1, equal amounts of seed were sampled from each half-sib family and planted in 72-cell trays to create the C3 population. In contrast to C1, all seedlings were planted in the field after tissue sampling to facilitate retraining of the initial GS model, and the intention was to obtain GEBVs prior to the flowering using M1, so that genomic selection could be conducted before flowering. However, a lag in genotyping prevented selections from being made in time for flowering. To allow selection on both parents, as was initially planned, cuttings were taken from selected plants and randomly mated in the greenhouse.

Selection in C3 proceeded in the greenhouse in a similar fashion as C1. Unlike the previous two generations of GS, M2 was used for prediction.

***Genetic Gain Trials*** Field trials were conducted in the summers of 2017 and 2018 to evaluate gain from selection. In 2017, trials were held at three different sites (Field-EI1, Field-Fr1, and Field-Fr2), one of which was the same site used to train the initial GS model. A randomized complete block design (RCBD) with two intra-block replicates was used at all locations in 2017. Plot size and number of replications differed depending on field dimensions and available seed. Four reps were grown at Field-EI1 and three at Field-Fr1 and Field-Fr2. Two plots per block each consisting of eight, nine, and ten plants were used at Field-EI1, Field-Fr2, and Field-Fr1 respectively. Only one site, Field-F3, was used in 2018 and a standard RCBD without intra-block reps was used. This site included three reps of 20-plant plots. The population C2 was only included in two sites in 2017 and was not included in 2018 due to seed yield. For the same reason, the C3 population was excluded from gain trials. Thus, all sites included at least the base population (C0), the population resulting from the initial round of phenotypic selection (C1), and the final population resulting from three rounds of GS (C4). Two sites included an intermediate population resulting from one round of GS (C2). The two parents of the population and the F1 were included in each block as checks in both years.

### Statistical methods

***Estimation of Parameters*** All available data were pooled to estimate phenotypic correlations ($r_{p}$), trait repeatabilities (*t*), genetic correlations ($r_{g}$), and trait narrow-sense heritabilities ($h^{2}$). Unless otherwise noted, all calculations were done in the statistical computing environment R (R Core Team 2019) and mixed models were fit with “ASRemlR v3” (Butler *et al.* 2009) for selection and parameter estimation.

As data were from three different environments, phenotypic records were regressed against environment and the residuals were used for calculating the Pearson correlation between traits. Estimates of $r_{p}$ were assessed for significance using a *t*-test, as implemented in the R lm() function.

Since multiple fruit per plant were measured for each trait, except for yield-related traits, it was possible to calculate *t* and $h^{2}$ using a genetic repeatability model:

y=Xβ+Zμ+Zp+e (1)

The parameters *μ* and *p* are random genotype and permanent environment effects respectively, and it is assumed that $\mu ∼N\left(0,\sigma_{u}^{2}K\right)$ and $p∼N\left(0,\sigma_{p}^{2}I\right)$. A so-called permanent environment effect is fit when repeated measures are obtained on the same individual (*i.e.*, multiple fruit on the same plant) and allows for partitioning of variation within and between genotype (Mrode 2014). The matrix X is the design matrix for *β*, which included a fixed effect for environment. Variance components estimated from this model were then used to calculate *t* and $h^{2}$ as follows (Falconer and Mackay 1996):

$$t=\frac{\sigma_{u}^{2}+\sigma_{p}^{2}}{\sigma_{u}^{2}+\sigma_{p}^{2}+\sigma_{e}^{2}};\;h^{2}=\frac{\sigma_{u}^{2}}{\sigma_{u}^{2}+\sigma_{p}^{2}+\sigma_{e}^{2}}$$ (2)

In these equations $\sigma_{u}^{2}$ is an estimate of additive genetic variation, $\sigma_{p}^{2}$ is a permanent environment effect related to variation within a genotype, and $\sigma_{e}^{2}$ is residual error. The gain in accuracy (Δr) from repeated measures given *t* was calculated as (Mrode 2014):

$$\text{Δ}r=\sqrt{\frac{1}{t+\frac{\left(1-t\right)}{n}}}$$ (3)

The heritabilty of the index used for selection was calculated using the formula for the variance of a linear combination of random variables (Lynch and Walsh 1998):

$$h_{I}^{2}=\frac{b^{T}Gb}{b^{T}Pb}$$ (4)

The matrices G and P are genetic and phenotypic covariance matrices for component traits of the index, and *b* is a vector of index weights.

Heritability estimates were used to calculate the expected accuracy of phenotypic mass selection. From theory, a regression of true breeding value on phenotype yields a correlation equal to the square root of $h^{2}$ (Falconer and Mackay 1996). Thus, for a given trait, the maximum predictive ability (PA) expected from phenotypic selection would be the square root of the trait’s true narrow sense heritability ($PA_{max}=h$). This value was used as a benchmark for our whole-genome regression models.

Genetic correlations were calculated for each trait pair using variance components estimated with a bivariate model. The mean of single-fruit measurements was used as the phenotypic value for each genotype. As with the repeatability model, a fixed effect was included in the bivariate model for environment, fitting a separate fixed environment effect for each trait. To test the significance of genetic covariances, a reduced model was fit specifying a diagonal matrix for the trait genetic covariance matrix (G) and compared to the full model, which was fit with an unstructured G, using a log-likelihood test.

Many pair-wise significance tests were conducted when testing phenotypic correlations and genetic covariances. In order to reduce false positives, p-values were adjusted with the Bonferroni method using the R function p.adjust().

***Cross-validation*** Marker sets for cross-validation (CV) were created by independently filtering each data set as previously described. Data sets were then joined on common markers to produce CV sets, which are shown in Table 2. For traits measured on a single-fruit basis, the truth-value used for CV was the mean of multiple fruit per plant. Since plants differed in number of fruit sampled, we tested the use of weights to account for heterogeneous error variance. Suitable weights for a model using the mean of repeated records can be obtained as:

**Table 2:** Cross-validation scenarios

Set Name	Schema	Training Size	Validation Size	Marker Number
CV_C0_	C0↻	122	31	130
CV_C2_	C2↻	132	34	1951
CV_T1_	T1↻	136	35	3307
CV_Strat_	(C0,C2,T1) → (C0,C2,T1)	48-402	75	535
Prog	C0 → C2	179	168	535
Test	C0 →T1	179	172	535

Summary of different cross-validation (CV) methods used in this study. Within-population schemes were tested in C0 (CV_C0_), C2 (CV_C2_), and in the test population (CV_T1_). Across population testing included predicting progeny from C0 (Prog) and T1 from C0 (Test). A stratified approach (CV_Strat_) was used to determine the role of population size on prediction accuracy.

$$w=\frac{1-h^{2}}{ch^{2}+\frac{1+\left(n-1\right)t}{n}-h^{2}}$$ (5)

where *t* is trait repeatability, $h^{2}$ is heritability, *n* refers to number of measurements, and *c* is the proportion of variance explained by marker data (Garrick *et al.* 2009). Several values of *c* were tested from 0.1 to 0.9. The use of weights was found to have a negligible effect on cross-validation results, so only results from unweighted analysis are given. The Pearson correlation between GEBVs for individuals with masked trait values and their corresponding truth-value was used as an estimate of model PA. Three distinct scenarios were tested: within-set CV, across-set CV, and a stratified CV approach.

Within-set PAs are commonly reported as an indication of model performance (Heffner *et al.* 2010). For within-set validation, a train-test approach was used. The data were randomly partitioned so that 80% of the data were used to train the prediction model and the remaining 20% were used to test the model. This process was repeated fifty times for each trait in each set (CV_C0_, CV_C2_, CV_T1_). The same random subsets were used across traits to enable comparison of PA for different traits.

Across-set CV represents a more realistic scenario than within-set CV; the model is used to predict either progeny derived from the training set (Prog) or individuals closely related to the training set, not just individuals that are a subset of the training set (Test). Both cases were tested by using a model trained in C0 to predict progeny in C2 (Prog) and to predict close relatives in T1 (Test).

The effect of training population size on model PA was investigated using a stratified CV approach (CV_Strat_). Briefly, data from C0, C2, and T1 were combined and different-sized training sets were formed by drawing an equal number of individuals from each of the three sets. The resulting composite set was used for training and then to predict a test set created by randomly sampling twenty-five of the remaining individuals from each set. CV was carried out fifty times for each population size-trait combination. Using a stratified scheme prevented any imbalance in the training or testing set that may have otherwise led to bias in assessing how PA changes with training population size, *e.g.*, if only individuals from C0 were used for training and only individuals in C2 were used for testing.

***Model for Genome-wide Selection*** A multi-trait GBLUP model with three traits, ${}^{\circ }$Bx, %DM, and a*, was used to calculate GEBVs for selection candidates. The multi-trait GBLUP model has the form:

y=Xβ+Zμ+e (6)

where *y*, *μ*, and *e* represent vectors ${\left(\begin{array}{c} y_{1}{}^{\prime },y_{2}{}^{\prime },y_{3}{}^{\prime } \end{array}\right)}^{\prime }$, ${\left(\begin{array}{c} \mu_{1},\mu_{2},\mu_{3} \end{array}\right)}^{\prime }$, and ${\left(\begin{array}{c} e_{1}{}^{\prime },e_{2}{}^{\prime },e_{3}{}^{\prime } \end{array}\right)}^{\prime }$ respectively. The values in *y* correspond to individual fruit measurements from each plant. A repeatability model was not used. It is assumed that μ∼N(0,K⊗G) and e∼N(0,I⊗R). The matrices G and R are unstructured and specify the genetic and error covariances between traits.

Equal economic weights were used in the selection index. With equal weights, the selection index value *I* for each selection candidate is given by:

I=GEBVB∘x+GEBV%DM+GEBVa* (7)

***Gain from Selection*** Realized gain from selection in the index and for other traits was determined using the following mixed-model fit with the ASReml standalone program (Gilmour *et al.* 2015):

$$y_{ijkl}m=\mu +s_{i}+b_{j\left(i\right)}+p_{k\left(j\right)}+c_{l}+e_{ijkl}m$$ (8)

In this model, *μ* is an overall mean, $s_{i}$ refers to a random effect for site (unique location x year), $b_{j\left(i\right)}$ is a random block effect nested in site *i*, *p* is a random effect for plot nested within block, *c* is a fixed effect covariate for cycle of selection, and *e* is a random error term. *b* (block) and *p* (plot) were modeled as heterogeneous random effects allowing different variances at each site. Plots within blocks (intra-block reps) were only included in 2017 and so *p* was only fit at the levels for those sites. *c* was coded as covariate 1,2,3 or 5, where 1 is the base population and the last cycle of selection is 5. The estimate of *c* is a slope corresponding to the average gain per cycle of selection. The significance of this slope (test for non-zero) was assessed using the p-value from the Wald F statistic calculated by the default method in ASReml.

As we did not genotype entries in the gain from selection trials we could not fit a MT-GBLUP model to calculate the index as we did during selection, which was the sum of BLUPs from the multi-trait model. Instead, we calculated the index value by applying weights to the phenotypes according to the Smith-Hazel index (Baker 1986):

$$b=P^{-1}Ga$$ (9)

where P and G refer to phenotypic and genetic covariance matrices calculated as described in the section on parameter estimation, *a* is a vector of ones to signify a equal economic weights, and *b* is the vector of weights applied to the phenotypic values to get the index value.

Harvesting practices differed somewhat across sites depending on whether vines could be separated before harvest. Either all fruit were harvested from a plot or all plants within a plot were harvested keeping track of single plants. In the former case, *y*s are means of all fruit in a plot, and in the latter *y*s are means of plant values for a plot, which are taken as the mean of all fruit from a plant. To account for this difference, the error variance was modeled as heterogeneous with sections corresponding to each case. Site and year are confounded in this design as there was no site used in both years.

### Data availability

Raw sequencing data from each population are available on the NCBI SRA. The BioProject reference ID is PRJNA611090. Phenotype data used to train selection models, the data from the realized gain trials, and all other data and code used in this study are available on GitHub (https://github.com/ch728/squash-gs). Supplemental material available at figshare: https://doi.org/10.25387/g3.11955216.

## Results

### Phenotypic and genetic parameters

All repeatability (*t*) estimates were moderate to high (see Table 3). Based on equation 3, measuring around 4-6 fruit per genotype is sufficient to realize most of the benefit from repeated measures for the range of repeatabilities observed in this experiment (see Figure S1). Repeatability sets the theoretical upper limit for $h^{2}$ (Falconer and Mackay 1996). Fitting within this constraint, estimates of $h^{2}$ were less than corresponding repeatability estimates. In most cases, $h^{2}$ estimates were substantially lower than repeatability estimates; fruit are relatively uniform within plants, but environment is a main driver of between-plant variation. The low to moderate heritabilities observed across traits underscores the quantitative nature of fruit traits in squash. Fruit morphology traits had the highest heritability estimates (0.27 - 0.38), while those for fruit quality and yield traits were the lowest (0.10-0.23). Color-related traits had the highest heritability among the quality traits, and total fruit count was twice as heritable as the other two yield measurements. The last column of Table 3 shows the maximum predictive ability (PA) expected for phenotypic mass selection. In the best-case scenario, it is expected that GS model PAs—if they can achieve at least the accuracy of phenotypic mass selection—should be moderate for quality and yield traits, and higher for morphology traits.

**Table 3:** Parameter estimates from pooled data

Trait	$h^{2}$	*t*	$PA_{max}$
a*	0.23	0.62	0.48
b*	0.18	0.55	0.42
${}^{\circ }$Bx	0.10	0.45	0.31
%DM	0.13	0.51	0.36
L*	0.18	0.55	0.42
Len	0.38	0.61	0.62
Wd	0.27	0.42	0.52
Wt	0.27	0.45	0.52
Shp	0.37	0.58	0.61
FrtCt*^a^*	0.20	—	0.45
TotalWt*^a^*	0.12	—	0.35
TotalDM*^a^*	0.11	—	0.33
*I*	0.35	—	0.59

a *t* is not reported for traits based on single measurements.

As shown in Table 4, all traits were significantly phenotypically or genetically correlated with at least one other trait. Traits were more strongly correlated within class than between classes, *i.e.*, quality traits had higher association with other quality traits than with yield or morphology traits. High association at the phenotypic level almost always translated to being highly associated at the genetic level as well.

**Table 4:** Phenotypic and genetic correlations; genetic correlations are shown above the diagonal and phenotypic correlations below^a^

	Fruit Quality					Morphological				Yield		
Trait	a*	b*	L*	${}^{\circ }$Bx	%DM	Len	Wd	Wt	Shp	FrtCt	TotalWt	TotalDM
a*		0.48*	−0.63**	0.24	0.32	−0.066	−0.12	−0.23	0.016	0.21	−0.17	−0.056
b*	0.35**		−0.34	0.58*	0.64*	0.024	0.032	−0.069	0.014	0.067	0.011	0.36
L*	−0.69**	−0.26**		−0.16	−0.12	0.29	0.3	0.41*	0.082	−0.035	0.26	0.15
°Bx	0.51**	0.31**	−0.36**		0.93**	−0.22	0.18	0.21	−0.28	−0.26	−0.4	0.11
%DM	0.37**	0.48**	−0.16**	0.76**		−0.04	0.042	0.067	−0.046	−0.035	−0.24	0.23
Len	0.0094	0.0069	−0.02	−0.051	−0.015		0.36*	0.52**	0.77**	−0.3	0.13	−0.049
Wd	−0.079	0.09*	0.071	−0.013	−0.038	0.33**		0.91**	−0.32*	−0.59*	0.34	0.29
Wt	−0.033	0.19**	0.037	0.065	0.07	0.51**	0.83**		−0.11	−0.69**	0.25	0.21
Shp	0.065	−0.058	−0.073	−0.034	0.014	0.73**	−0.39**	−0.1*		0.16	−0.1	−0.22
FrtCt	−0.11*	−0.069	0.14*	−0.16**	−0.14**	−0.053	−0.14*	−0.2**	0.04		0.45	0.42
TotalWt	−0.11*	0.03	0.12*	−0.11*	−0.098*	0.18**	0.25**	0.27**	−0.017	0.84**		0.88*
TotalDM	0.036	0.22**	0.04	0.19**	0.28**	0.17**	0.24**	0.3**	−0.014	0.74**	0.91**	

a Two levels of significance are reported: significance at a p value of 0.05 (*) and significance at a p value of 0.05 after Bonferroni multiple test correction (**). Significance of genetic correlations were not tested directly; the significance designation indicates that allowing genetic covariance between the two traits significantly improved the fit of the bivariate model.

Within quality traits, ${}^{\circ }$Bx and %DM had the highest phenotypic and genetic correlation. All quality traits were either correlated in a favorable direction or not significantly correlated. However, there were trade offs between quality traits and traits from other categories. For example, almost all of the fruit quality traits had a unfavorable genetic correlation with total fruit weight per plant. Most of the correlations between quality and morphology traits were antagonistic, which reflects a tendency for smaller fruit to have more favorable quality characteristics.

### Model predictive ability

**Figure 2:**
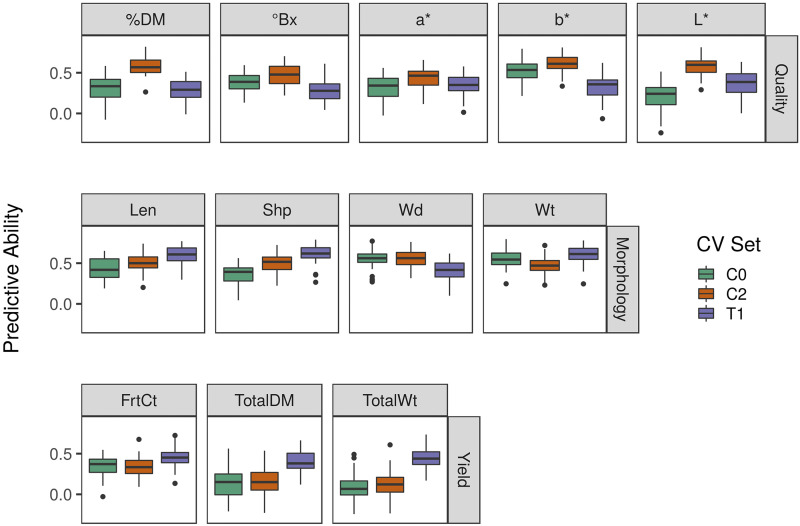
Cross-validation results. Boxplots show the predictive ability for each trait grouped by cross-validation set and trait type. Cross-validation was conducted within the base population (CV_C0_), the C2 population (CV_C2_), and in the test population (CV_T1_).

Whole-genome models were evaluated for all traits. As shown in Figure 2, within-set PAs (CV_C0_, CV_C2_, and CV_T1_) were low to moderate for most traits, mirroring heritabilites. Yield component traits such as FrtCt and TotalWt were the most difficult to predict, whereas, models for morphology traits tended to have higher PA. There was a high variance observed for predictive abilities. This is likely due to the small size of the sets being subsampled to estimate predictive ability.

PAs from the Across-set CV schemes (Prog and Test) are given in Table 5. In general, the PAs in either set were within the range of PAs observed in the within CV sets, typically close to the mean. Within-set PAs were often more optimistic than the PAs observed across sets. There was not a clear pattern as to whether a trait would be better predicted in the Prog or Test set. As both sets are highly related to C0, some of these differences may be due to genotypic x environment (G x E) interactions.

**Table 5:** Cross-validation results for Test and Prog schemes

Trait	Prog	Test
a*	0.33	0.29
b*	0.47	0.14
${}^{\circ }$Bx	−0.02	0.31
%DM	0.44	0.27
L*	0.48	0.25
Len	0.32	0.54
Wt	0.41	0.57
Wd	0.48	0.39
Shp	0.38	0.52
FrtCt	0.21	0.34
TotalWt	−0.13	0.15
TotalDM	0.10	−0.03

**Figure 3:**
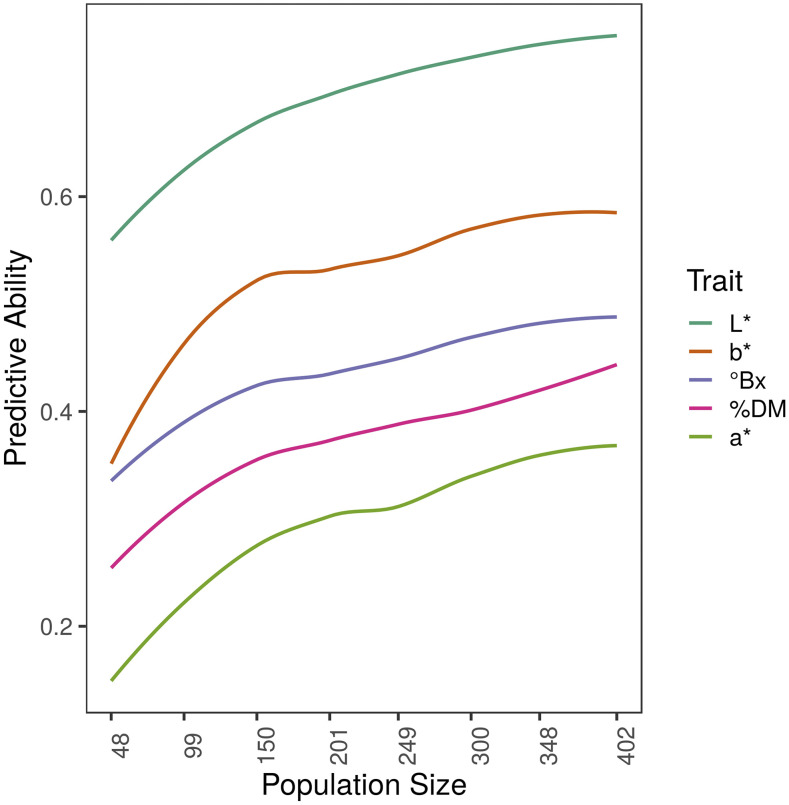
Effect of population size on predictive ability determined using CV_strat_ scheme.

Figure 3 shows results from the stratified CV scheme. Increasing training population size increased the PA for all traits, but the gain was not substantial for most traits. There was little benefit to having a training population size beyond 200 plants. This has been observed in other studies examining PA in biparental populations (Heffner *et al.* 2011).

### Gain from selection

**Figure 4:**
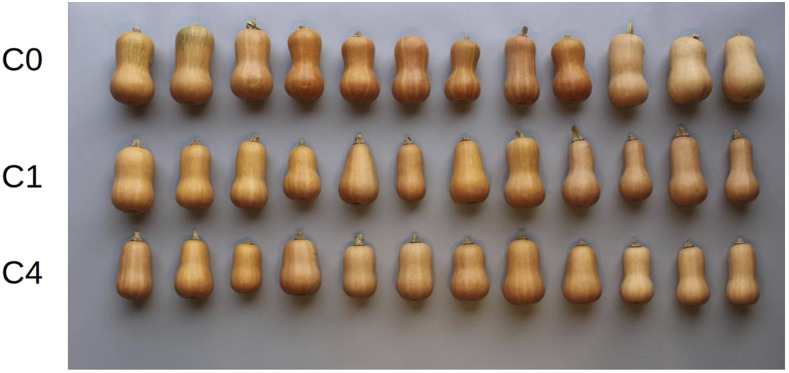
Fruit from selection cycles.

Results from the selection experiment indicate significant change (p<0.001) in all traits under selection. The index value and all component fruit quality traits were increased in the desired direction. The traits a*, ${}^{\circ }$Bx, %DM, and I increased by 0.76, 0.40, 0.82, and 0.50 units per cycle on average. Along with a*, there was a significant correlated response in the other two color traits, b* and L*, indicating that flesh of selected fruit were darker orange than unselected fruit. Morphology traits exhibited a slight, but significant, correlated response from selection; selection for increased fruit quality lead to a slight enrichment for smaller-sized fruit. A summary of gain from selection is given in Table S1. A representative sample of fruit from different cycles of selection are pictured in Figure 4.

## Discussion

In this paper, we present estimates for several important quantitative genetics parameters including heritability and genetic correlations for fruit traits in butternut squash, an important market class of *C. moschata*. Traits encompassed quality-related traits, such as ${}^{\circ }$Brix and percent dry matter, morphological characteristics, and several different components of yield. Along with estimating these parameters, we tested the predictive ability of genome-wide models (GBLUP) for each trait across several different testing scenarios. Additionally, results are presented from a selection experiment that was conducted using an index estimated from a multi-trait GBLUP model aimed at improving Brix, % dry matter, and a*.

Repeatability estimates were found to be moderate across traits. Narrow-sense heritability estimates were considerably lower. As squash plants can yield many fruit, there is a labor trade-off between the number of fruit sampled per genotype and replication of the genotype itself. The discrepancy between repeatability and narrow sense heritability suggests that more resources should be allocated to replicating a genotype rather than sampling more fruit per genotype. The range of repeatabilities observed for fruit traits suggest little benefit to measuring more than 4-6 fruit per plant. Genetic and phenotypic correlations were high between many of the traits measured in this study. Quality traits were found to be favorably associated with other quality traits—an ideal situation from a breeding perspective. However, quality traits were unfavorably correlated with some morphology and yield components. Most of these correlations were low, which means quality need not come at the expense of yield. It is important to consider that genetic correlations are not permanent, but are driven by pleiotropy and linkage, both of which can be manipulated either directly or indirectly by the breeder (Falconer and Mackay 1996).

Genetic correlations mirrored phenotypic correlations for many traits, but there were some instances where this was not the case. For example, a* and Brix had a strong and significant phenotypic correlation (0.51), but they had a weak and non-significant genetic correlation (0.24). Phenotypic correlations are a product of both genetic and error correlation; two characteristics can have high association at the phenotypic level due to common effect of the environment without being highly related at the genetic level (Baker 1986). This is likely the case for Brix and a*. In contrast, Brix and % dry matter were highly correlated at both levels. Brix is used in programs as a proxy for sweetness, and % dry matter is related to starchiness (Corrigan *et al.* 2001; Loy 2004). As the starch and sugar pathways are known to be interrelated, it is not suprising that Brix and % dry matter would have high estimated genetic correlations (Wyatt *et al.* 2016).

Parameters estimated in this study are not likely to generalize to all winter squash populations. All parameters are specific to a population, the way in which traits are measured, the environments sampled, and are also subject to error in estimation (Hallauer and Miranda 1981). This being said, they still give a general sense of trait complexity. For example, the low estimates of $h^{2}$ for quality traits suggests a highly quantitative nature for these traits which is likely to be the case in many other squash populations.

The PA of GBLUP was explored for a wide assortment of fruit traits across several different CV schemes. For the most part, model PA for each trait was correlated with trait narrow-sense heritability. Some traits, such as %DM, exhibited higher than expected PAs. This does not indicate a problem with the model, but rather, suggests some imprecision in the estimate of $h^{2}$. This can occur when GBLUP is used to estimate narrow-sense heritability (de Los Campos *et al.* 2015). The mean PA across different CV sets was similar for most traits. Some traits including TotalWt, b*, and ${}^{\circ }$Bx exhibited more variation in PA across sets. This may be partially due to G x E, which has been noted for ${}^{\circ }$Bx and color-related traits in squash (Paris 1994; Harvey *et al.* 1997). The same filtering procedure was applied to all marker sets, but marker number varied across sets due to differences in data quality and sites sampled during sequencing—problems intrinsic to GBS. Therefore, some of the differences in PA across sets may be due to different regions of the genome being sampled; although, the high levels of relatedness in an F2 likely reduces the impact of having different marker numbers across sets (Habier *et al.* 2007). The Prog and Test sets represent scenarios where models are used to predict the progeny of the training population or a highly related set, which are common use cases for GS (Heslot *et al.* 2015). PAs were similar to the means of the PAs observed in the within sets.

Selecting for multiple traits is a challenge impacting all breeders. There are three general methods for multi-trait selection: tandem selection, independent culling, and index selection (Baker 1986). Index selection requires all traits to be measured on all individuals, which is not required by the other two methods. This gives other multi-trait selection methods a potential economic advantage because selection can begin with the least expensive trait and follow with culls on progressively fewer individuals for more costly phenotypes. GS can reduce this disadvantage by relaxing the need to phenotype all individuals; expensive traits can be predicted from marker data rather than directly measured. Further, MT-GBLUP models can lead to higher PAs in some scenarios and allow for the estimation of parameters needed to implement an optimal index, such as a Smith-Hazel index (Okeke *et al.* 2017; Rutkoski *et al.* 2016).

In this study we used MT-GBLUP to select on an index of three fruit quality traits. Our index used equal economic weights. Significant gain was achieved in all quality traits and in the index value. Significant changes were also observed in correlated traits. This emphasizes the need to understand the relationship between traits, as unintended consequences may result from selection on some characteristics. We used equal economic weights, as we had no way of readily establishing the worth of quality traits in our study. A possible strategy for establishing relative weights for quality traits may be to use a retrospective index (Bernardo 1991). By analyzing the past selections of an experienced breeder or selections that a trained panel of tasters/chefs make, one could derive retrospective weights and use them as relative economic weights to create an index.

Simulation has demonstrated that one of the greatest advantages of GS over phenotypic selection comes from the ability to decrease cycle length with GS, even if this comes at the expense of accuracy (Gaynor *et al.* 2017). We were able to achieve significant gain in spite of the fact that our model had relatively low PAs for some traits included in the index, and continued rapid recycling would likely outperform the traditional phenotypic selection program; although, we did not test our GS program head-to-head with the traditional phenotypic selection program. Unlike cereal crops, squash and other large-fruited crops are not limited by the need for seed increase at early stages—sufficient seed can be obtained from fruit to allow testing across multiple environments at an early stage in the breeding program. However, large replicated trials are not feasible due to space constraints. Markers can be used to enable unbalanced designs in early testing stages and could potentially be used to evaluate more material in small trials across multiple different environments (Endelman *et al.* 2014). This sparse testing approach would likely be beneficial to space-limited crops like squash. Further, this approach would be an easy entry point for programs that are new to using whole-genome marker data, as no major changes would be made to the breeding scheme. Additionally, it would allow programs to build up large training sets, and work out the logistic and technical aspects of genotyping many entries in a timely manner, before transitioning to a rapid-cycling scheme.

## Conclusion

We presented results demonstrating the successful implementation of GS in a resource-limited crop for several fruit quality characteristics. Further, prediction results were shown for many other traits and several important quantitative genetic parameters were calculated. These results were used to suggest different breeding strategies.

It can be argued that some traits can only be judged by the human eye, especially in horticultural crops where there is more potential overlap between art and science. GS need not subtract from this aspect of breeding horticultural crops. GS can be used to enrich populations for desirable characteristics and to improve breeding efficiency at early stages in the breeding process where there is a glut of inferior material. MT-GBLUP and indices provide a convenient means of integrating information for the improvement of multiple traits at these stages. This leaves the breeder with more time to focus on subjective evaluations, like culinary testing, on only the best material at advanced stages of variety testing.
